# Pentylenetetrazole kindling impairs place recognition memory associated with suppressing proBDNF-mediated neural information flows at the hippocampal CA3-CA1 synapses

**DOI:** 10.3389/fphar.2026.1881525

**Published:** 2026-07-15

**Authors:** Wei Sun, Zhanyong Li, Yang Yang, Yang Chen, Xiaoliang Li, Chundan Zhang, Lei An

**Affiliations:** 1 Department of Emergency, The First Affiliated Hospital of Guizhou University of Traditional Chinese Medicine, Guiyang, China; 2 Department of Neonatology, The First Affiliated Hospital of Guizhou University of Traditional Chinese Medicine, Guiyang, China; 3 School of Life Sciences, Langfang Normal University, Langfang, China; 4 Department of Neurology, Jinan Geriatric/Rehabilitation Hospital, Jinan, China

**Keywords:** epilepsy, hippocampal CA1, information flow, neural activity, proBDNF

## Abstract

**Introduction:**

Mature brain-derived neurotrophic factor has long been known to as an epigenetic regulator. It is excessively secreted after status epilepticus, leading to a variety of permanent structural and functional changes in the brain. While its precursor forms proBDNF regulates nerve development, neural transmission and cognitive function, it is not fully known whether the expression of proBDNF is changed *in vivo* or how it influences neuronal signaling and function ultimately leading to spatial cognitive impairments.

**Methods:**

Here, we investigated changes in proBDNF levels in the hippocampus of the pentylenetetrazole (pentylenetetrazole)-induced epileptic rat model. Functional blocking of proBDNF singling and its related pathway in the hippocampal regions was conducted to explore the potential mechanisms. Meanwhile, local field potentials were recorded in the hippocampal CA3-CA1 pathway and the directionality of neural information flow (NIF) between two regions was evaluated.

**Results:**

We found that seizures were induced by PTZ-treated rats, which exhibited excessive proBDNF expression in the hippocampus only after undergoing behavioral training. Intra-hippocampal infusions of anti-proBDNF antibody into the CA1 but not the CA3 region could mitigate the PTZ-induced memory consolidation deficits and we confirmed the involvement of p75^NTR^ rather than TrkB signaling. The excessive proBDNF could act on both presynaptic and postsynaptic sites through p75^NTR^ signaling to exaggerate neural activity of putative fast-spiking interneurons. This was evidenced by increased spontaneous excitatory postsynaptic current frequency and amplitude, and further corroborated by action potential-independent miniature EPSC (mEPSC) recordings, which revealed concurrent increases in both mEPSC frequency and amplitude specifically in the epilepsy group. Importantly, this over-expression of proBDNF disrupted phase synchronization and directional coupling strength at the CA3 to CA1 synapses. However, blocking proBDNF or inactivation of the p75^NTR^ signaling could effectively enhance the phase-locked value and neural information flow at the gamma and high-frequency oscillations, and significantly alleviate the PTZ-induced impairments in memory processing.

**Conclusion:**

Our findings are consistent with the hypothesis and provide the first direct evidence that the over-activation of proBDNF signaling represents a potential mechanism involved in neural dysfunction and NIF disruption leading to memory impairments in kindled animals.

## 1 Introduction

Epilepsy, defined by recurrent seizures resulting from an imbalance between cerebral excitability and inhibition, is a major complex neurological disorder affecting approximately 50 million individuals worldwide (Cano et al., 2021; Serratto et al., 2020; Shore et al., 2020). Morphological, molecular, and functional alterations in epileptic tissue induce abnormal neuronal responses to sub-threshold stimuli or spontaneous activity, marked by paroxysmal, high-amplitude spike-and-wave discharges (Frolov et al., 2019; Bonaccini Calia et al., 2022; Komoltsev et al., 2023). Consequently, firing of individual neurons or small neuronal populations can trigger synchronous action potential propagation and neural network disruption, ultimately contributing to cognitive impairments (Pototskiy et al., 2021; Arski et al., 2022; Shahpari et al., 2022).

Mature brain-derived neurotrophic factor (BDNF) has long been recognized as an epigenetic regulator implicated in central nervous system (CNS) development, synaptic plasticity, and long-term memory consolidation (Chen and Chen, 2017; Karpova, 2014; Miao et al., 2020). The mature form of BDNF is initially synthesized as a precursor protein (proBDNF), which exerts opposing effects on synaptic transmission and neural excitability (Yang et al., 2014; Gibon et al., 2015). Recent studies have demonstrated that entorhinal proBDNF and its receptor p75^NTR^ regulate neural network homeostasis and modulate behavioral outcomes (Sun et al., 2022a; An et al., 2018; Ma et al., 2022). For instance, inactivation of the proBDNF-p75^NTR^ signaling pathway in mice led to improved working memory but heightened susceptibility to severe seizures (Gibon et al., 2015). While epileptic seizures upregulate mBDNF expression (Sharma et al., 2020; Kazmi et al., 2020; Egbenya et al., 2023), other investigations have indicated that intra-hippocampal infusion of mBDNF or TrkB receptor activation confers enhanced resistance to kindling and protected against epileptogenesis (Falcicchia et al., 2018; Iughetti et al., 2018; Gu et al., 2022). These conflicting observatons may partly stem from the distinct roles of proBDNF and mBDNF in epileptogenesis (Gibon et al., 2016; Holm et al., 2009). Moreover, the potential involvement of pro-neurotrophins in epileptogenesis is supported by evidence that enhanced cleavage of pro-nerve growth factor (proNGF) to produce mature NGF elicits neuroprotection following kainate administratin in organotypic slice cultures (Le and Friedman, 2012). Notably, recent research has identified epileptic influences on the regulation of activity-dependent *Bdnf* gene transcription through DNA methylation mechanisms during the long-lasting memory formation (Parrish et al., 2015; Martinez-Levy et al., 2018; Skupien-Jaroszek et al., 2021). Crucially, elevated proBDNF expression post-training is necessary for memory formation via activation of postsynaptic receptors (An et al., 2018; Sun W. et al., 2019; Sun et al., 2018a). Indeed, proBDNF signaling engages multiple downstream protein kinases to activate target proteins, resulting in enhanced presynaptic mediator release or modification of postsynaptic receptors activity that mediate inhibitory neurotransmission and balance neural network excitation (Sun et al., 2022a; Sun W. et al., 2019; Sun et al., 2020). Although the involvement of mBDNF in epileptogenesis was established, the expression patterns and functional impacts of its precursor proBDNF have not yet been fully elucidated.

Numerous reports have indicated that BDNF levels were elevated in the hippocampus after seizures induced by pentylenetetrazole (PTZ) (Sharma et al., 2020; Kazmi et al., 2020), pilocarpine (Yu and Jiang, 2020) and kainite (Egbenya et al., 2023). The hippocampus serves as a critical structure for spatial learning and memory in mammals (Anderson et al., 2014; Moser et al., 2017). Learning-induced modulation of regular-spiking (RS) pyramidal neurons and fast-spiking (FS) interneurons in the hippocampus is thought to underlie a mnemonic mechanism involving intrinsic and network components (He et al., 2023; Khan et al., 2018; Nicole et al., 2016; Li et al., 2022). Furthermore, neuronal oscillations transmit information from the external environment to the hippocampus, and also carry information across large regions of the cerebral cortex. The process is mediated by electronic spiking to enable rapid integration of information processing (Amemiya and Redish, 2018; Asgeirsdottir et al., 2020; Chung et al., 2021; Rothschild et al., 2017). The fast rhythms including gamma and high-frequency (HF) bands are mediated by BDNF signaling and are primarily observed in the CA1-CA3 system. These rhythms are likely involved in spatial long-term memory and cognitive task performance (Sun et al., 2018b; Huang and Morozov, 2011; Tamura et al., 2018; Jones et al., 2017). Moreover, proBDNF secretion at synapses can regulate these beta and gamma rhythms through p75^NTR^ that drives the shift of neuronal responses from excitation to inhibition (Sun et al., 2022a; Langlois et al., 2013; Nagappan et al., 2009). Additionally, theta, beta and gamma bands in the amygdala and thalamocortical circuits are involved in neurological disorders (Khastkhodaei et al., 2021; Ghosh et al., 2013), which include epileptiform activity (Aupy et al., 2019; Rehulka et al., 2019; Tamrakar et al., 2022).

To better understand proBDNF potential role in epilepsy, we monitored hippocampal levels of proBDNF and mBDNF in a PTZ-induced kindling animal model. Pharmacological infusions were employed to distinguish and characterize effects on memory phases, hippocampal regions and signaling pathways. Furthermore, excitatory postsynaptic currents (EPSCs) were recorded to assess potential neural mechanisms. To definitively isolate pre- and postsynaptic components of the observed synaptic changes, we also performed recordings of miniature EPSCs (mEPSCs).​ Finally, considering the training-specific changes in the hippocampal cells, we recorded the neural activity and neural information at CA3-CA1 synapses when animals were performing memory tests. These studies provide new insights into how proBDNF signaling contributes to memory impairments and neural dysfunction linked to epilepsy disorders.

## 2 Experimental procedure

### 2.1 Experimental animals

Male Sprague-Dawley rats were purchased from Sibeifu (Beijing) Biotechnology Co., Ltd. They were housed in plastic cages in a colony room maintained at 21 °C ± 2 °C; with 45% ± 5% humidity and a light cycle starting at 7:00. Rats were kept in transparent plastic cages with free access to food and water. All animal procedures complied with regulation from the Institutional Animal Care and Use Committee of Langfang Normal University (SCXK-2019-0010).

### 2.2 Animal kindling

As previously described, status epilepticus was induced via repeated intraperitoneal injections of pentylenetetrazole (PTZ). PTZ (37 mg/kg) was administered once every 2 days for a total of 14 injections and rats were monitored for 30 min post-injection each time (Hao et al., 2016; Sun Z. Q. et al., 2019; Cheng et al., 2019). Control rats received an equivalent volume of saline as vehicle controls. Seizure severity was scored based on the Racine scale (Racine, 1972). Briefly, phase 0 indicated no response. Phase one involved hyperactivity and vibrissae twitching. Phase two included head nodding head clonus and myoclonic jerks. Phase three was defined by unilateral forelimb clonus. Phase four consisted of rearing with bilateral forelimb clonus. Phase five referred to generalized tonic-clonic seizures with loss of postural control. Phase six indicated absence of movement and loss of vital organ function or mortality.

### 2.3 Behavioral tests

Behavioral testing was conducted in an open-field arena 60 cm long 60 cm wide surrounded by a 60 cm high white corrugated plastic wall equipped with visual cues. Hook and loop fasteners were placed to secure objects in four corners of the chamber during trials with each corner 10 cm from the two nearest sides. Prior to training rats underwent a 5 min habituation session daily for three consecutive days. No objects were placed in the chamber during habituation sessions though the fasteners remained attached to the floor. The objects used for testing were counterbalanced across experimental groups.

During training, rats were allowed to freely explore two identical objects in the chamber for 5 min. Object-place recognition (OPR) memory was tested 1 day after the training with one object moved to a novel location (Figure 3A). Between trials, the chamber and objects were cleaned with 70% alcohol to eliminate odor cues. During the 5 min memory test the ratio of time rats spent exploring the object in the novel location versus the familiar location (OPR ratio) was recorded. Total exploration time during both training and testing phases was also documented.

### 2.4 BDNF levels in the hippocampus

One day after the final PTZ injection or immediately after behavioral training, rats were deeply anesthetized and brains were removed following previously established protocols (An and Zhang, 2015; An and Zhang, 2013; An et al., 2012). Bilateral hippocampi were isolated on an ice-cold surgical platform. After weighing, each hippocampus was rinsed in RIPA buffer containing 50 mM Tris, 150 mM NaCl, 1 mM EDTA, 0.5% Triton X-100, 0.5% sodium deoxycholate (pH = 7.4) and a protease inhibitor cocktail. Mixtures were homogenized using a glass homogenizer for 5 min and centrifuged at 3,000 rpm at 4 °C for 15 min. The supernatant was collected and stored at −70 °C. Levels of mBDNF (Adipo Bioscience; Cat#SK00752-01) and proBDNF (Adipo Bioscience; Cat#SK00752-06) were quantified following protocols outlined in previous references using commercial ELISA kits. Optical density was measured at 450 nm with a microplate reader (Bio-Rad, United States). Sample protein concentrations were determined by the Coomassie Brilliant Blue G-250 method using bovine serum albumin as the standard.

### 2.5 Bilateral microinjection

Rats were anesthetized with isoflurane and prepared for surgery as previously reported (An et al., 2018; Sun et al., 2018a; Sun et al., 2021a). Stainless steel guide cannulae (22-Ga; Plastics One, Inc.) were bilaterally implanted to the hippocampal CA1 region (AP -3.5 mm, ML 2.5 mm, DV 2.0 mm) and a separate set was implanted into the hippocampal CA3 region (AP -4.2 mm, ML 3.5 mm, DV 2.5 mm). Obdurators (30-gauge, Plastics One Inc.) were inserted into the guide cannulae to prevent blockage. Immediately after surgery rats were treated with Anafen and allowed a recovery period of at least 7 days.

Microinfusions were performed using custom needles 30-Ga needles (Small Parts Inc.) connected to an infusion pump (Harvard Apparatus) via PE-50 tubing. Needles extended 1.0 mm beyond the tip of the guide cannulae. Either anti-proBDNF antibody (10 μg/μL; Cat#ANT-006, Alomone Labs), TAT-Pep5 (4 ng/μL; Cat#506181, EMD Millipore), K252a (25 μg/μL; Cat#82497; Sigma-Aldrich) or artificial CSF (ACSF, Cat#3525, Tocris Bioscience) was infused at a rate of 0.5 μL/min/side for 2 min. Infusions were administered immediately after training or 30 min before the memory test. Needles were left in place for 3–5 min to facilitate drug diffusion. For all intra-hippocampal pharmacological experiments, the ACSF served as the common vehicle control. In behavioral and electrophysiological tests, the control and epilepsy groups received ACSF infusions (0.5 μL/min/side for 2 min) into the CA1 region corresponding to the drug treatment, using the same procedure. One week before treatment, rats were habituated to the infusion procedure on two separate days. The same group of rats received all infused agents with assignments randomized and at least 1 week between consecutive injections.

### 2.6 Patch-clamp recordings

Rats were euthanized by decapitation and hippocampal slices were prepared following established methods (Sun et al., 2022a; Sun et al., 2022b; Sun et al., 2022c). Briefly, brains were removed and mounted in a chamber of a vibratome (VT1000S, Leica, Germany) to cut sequential coronal slices with a thickness of 300 μm. Slices were incubated in ice-cold (0 °C–4 °C) oxygenated (95% O_2_ and 5% CO_2_) ACSF containing 125 mM NaCl, 2.5 mM KCl, 1.25 mM NaH_2_PO_4_, 26.0 mM NaHCO_3_, 10.0 mM glucose, 2.0 mM MgCl_2_ and 2.0 mM CaCl_2_ (pH 7.3–7.4). Slices were maintained in continuously oxygenated ACSF at room temperature (approximately 22 °C) for at least 1 h before transfer to a controlled environmental chamber mounted on a contrast-enhanced CCD camera (Hamamatsu) with infrared gradient contrast.

Conventional whole-cell voltage-clamp recordings were performed on hippocampal putative FS interneurons using pipettes pulled with a P-97 electrode puller (Sutter Instruments). Putative fast-spiking (FS) interneurons were visually preselected under infrared differential interference contrast optics based on their somata morphology (typically small, round or ovoid cell bodies located in or near the pyramidal cell layer). Whole-cell recordings were established with the pipette interior solution and neurons were held at −40 mV to minimize the contamination of inhibitory postsynaptic currents. The FS phenotype of these neurons was further confirmed during online by the characteristically narrow waveform of action potentials that occurred spontaneously during the recording. Only neurons consistently exhibiting this property were included in the subsequent analysis of spontaneous excitatory postsynaptic currents (sEPSCs). Patch pipettes had a resistances of 4–5 MΩ when filled with an internal solution containing 135 mM K-gluconate, 0.1 CaCl_2_, 2.0 mM MgCl_2_, 10.0 mM HEPES, 2.0 mM MgATP, 0.3 Na_3_GTP, 0.2 EGTA and 4.0 Na_2_-phosphocreatine (pH 7.3). Using this internal solution, sEPSCs were recorded as inward currents. To investigate the contributions of proBDNF and neurotrophin receptors, the external solution was supplemented with either anti-proBDNF antibody (10 μg/μL; Cat#ANT-006, Alomone Labs) or the p75^NTR^ inhibitor TAT-Pep5 (4.0 ng/μL). All drugs were added during slice recovery and maintained throughout recordings. Data were collected using an EPC-10 patch-clamp amplifier (HEKA Instruments). Signals were digitized at 10 kHz, low-pass filtered at 2.9 kHz, and stored on a disk using Pulse software (HEKA, Germany). Neurons were excluded from analysis if series resistance varied by more than 10%. The detection threshold for EPSC detection was set at 5 pA. Spike waveforms were analyzed using Clampfit software (Molecular Devices).

To isolate action potential-independent quantal events, mEPSCs were recorded from a separate cohort of FS interneurons under identical experimental conditions, with the addition of 1 μM tetrodotoxin (TTX; Cat#T8024, Sigma-Aldrich) to the external ACSF to block voltage-gated sodium channels. Neurons were held at −70 mV. Recordings were performed for at least 5 min per cell to obtain a sufficient number of events for analysis. mEPSCs were detected and analyzed using the same threshold (5 pA) and software (Clampfit) as described for sEPSCs.

### 2.7 LFP recordings

Microelectrodes were configured into two 4 × 4 matrices using 25 μm diameter platinum/iridium wire coated with polyimide (California Fine Wire Company) and housed in a 16-gauge silica tube (World Precision Instruments) as previously described (Sun et al., 2018b; Sun et al., 2021b; Sun et al., 2022d). Electrodes were connected via gold pins to an EIB-36-PTB board (Neuralynx Inc.) assembled with a microdrive (Harlan 8-drive; Neuralynx). Electrode tips were gold-plated to maintain an impedance of 200–600 kΩ measured at 1.0 kHz (NanoZ Neuralynx). Rats were anesthetized with isoflurane and prepared for surgery to chronically implant two electrode arrays. One array was placed in the CA1 region (AP -3.5 mm, ML 2.5 mm, DV 2.0 mm) and the other one in the CA3 region (AP -4.2 mm, ML 3.5 mm, DV 2.5 mm). Implantation in the left or right hemisphere was randomized but counterbalanced across groups.

Recording was conducted using a Digital Cheetah system (Cheetah software, Neuralynx Inc.), sampled at 32 kHz and filtered between 0.1 and 9,000 Hz. Animals’ behavior was monitored with a digital ceiling camera (Neuralynx Inc.) and the camera’s signal was fed to a frame grabber (sampling rate 64 Hz) with experimental timestamps superimposed for offline analysis. A continuous 10-min neuronal firing recording was acquired immediately after behavioral training. Subsequently, each session began 5 min prior to the animal being placed in the behavioral arena and continued uninterrupted for the full 5-min duration of the memory test, resulting in a total LFP recording period of approximately 10 min per session per animal.

Unit activity was amplified 10,000 times, sampled at 32 kHz and filtered between 600 and 6,000 Hz. Offline spike sorting was conducted using SpikeSort 3D combining KlustaKwik with manual clusters adjustment (Klusters software package). Multiple parameters including spike height, trough, and energy were used to visualize the clustered waveforms. Units were assessed for quality and classified as putative regular-spiking (RS) pyramidal neurons or putative fast-spiking (FS) interneurons following methods described elsewhere (Sun et al., 2022d; Sun et al., 2021c; Sun et al., 2021d). LFPs were amplified 1,000 times, sampled at 32 kHz and filtered between 0.1 and 9,000 Hz from each electrode. Before analysis, DC offsets and slow fluctuations were eliminated using the locdetrend function in the Chronux 2.00 toolbox (Bokil et al., 2010), which subtracts a linear regression line with the following parameters 1-s window size 50-m time step 5 time-bandwidth product, and nine tapers. A Butterworth band-pass filter was applied to isolate frequency bands of interest including delta (0.5–3 Hz) theta (4–12 Hz) beta (13–35 Hz) gamma (52–100 Hz) and high-frequency (HF 100–250 Hz). Power spectral densities were calculated via fast Fourier transform based on Welch’s method with 1,024 frequencies between 1 and 200 Hz smoothed using a Gaussian Kernel with a 3-Hz bin width. All analyses were performed using Neuroexplorer and Matlab (MathWorks) software as reported in previous studies (Sun W. et al., 2019; Sun et al., 2020; Sun et al., 2021a).

### 2.8 Phase locked value (PLV)

The PLV is used to analyze the strength of phase synchronization. After extracting the phase of two signals, the respective phase values $\phi_{a}$ and $\phi_{b}$ were obtained. PLV is calculated as follows

$$PLV=\left|\frac{1}{N}\sum_{j=1}^{N}\exp\left(i\left[\phi_{a}\left(j\Delta t\right)-\phi_{b}\left(j\Delta t\right)\right]\right)\right|$$

with N representing the length of time series, $\frac{1}{\Delta t}$ is the sampling frequency. PLV values range between 0 and 1. A value of one indicates full synchronization while a value of 0 indicates no synchronization whatoever.

### 2.9 General partial directed coherence (gPDC) algorithm

PDC was developed to characterize causal relationships between multivariate time series. Its definition is based on the concept of linear Granger causality and its core principle relies on decomposing multivariate partial coherences derived from multivariate autoregressive models. The two-variate process PDC algorithm is descrbed as follows.

### 2.10 Considering a two dimensional process

$$X\left(t\right):=\left[x_{1}\left(t\right)\;x_{2}\left(t\right)\right]$$

Granger causality within the two-variate process defined by $X\left(t\right)$ is evaluated by modeling the process through a vector autoregressive (VAR) model with the form:

$$X\left(t\right)=\sum_{r=1}^{p}A_{r}X\left(t-r\right)+E\left(t\right)$$

with $A_{r}=\left[\begin{array}{cc} a_{11}\left(r\right) & a_{12}\left(r\right)\\ a_{21}\left(r\right) & a_{22}\left(r\right) \end{array}\right]$

Taking the Fourier Transformation of the VAR coefficient yields:

$$A\left(f\right)=\sum_{r=1}^{p}A_{r}\cdot \exp\left(-i2\pi fr\right)$$

which provides a frequency-domain representation of the VAR model.

Defining the matrix: $\overset{-}{A}\left(f\right)=I-\sum_{r=1}^{p}A_{r}\cdot \exp\left(-i2\pi fr\right)=\left[\overset{-}{a_{1}}\left(f\right)\;\overset{-}{a_{2}}\left(f\right)\right]$ as the difference between the identity matrix.

And then PDC from variable $x_{j}$ to $x_{i}$ is defined as:

$$\pi_{ij}\left(f\right)=\frac{{\overset{-}{a}}_{ij}\left(f\right)}{\sqrt{{\overset{-}{a}}_{j}^{H}\left(f\right){\overset{-}{a}}_{j}\left(f\right)}}$$

It has been shown that large differences in the variances of the modeled time series can lead to distortions in the resulting PDC values (Winterhalder et al., 2005; Baccala et al., 2007; Sun et al., 2021e). To avoid this, a variation of the original PDC which is called *generalized PDC* (gPDC) is presented (Baccala et al., 2007; Sun et al., 2022e; Sun et al., 2021f). In gPDC, the coefficients ${\overset{-}{A}}_{ij}\left(f\right)$ are normalized by the standard deviation of the $E\left(t\right)$ model residuals:

$${\pi_{ij}}^{g}\left(f\right)=\frac{\frac{1}{\sigma_{i}}{\overset{-}{a}}_{ij}\left(f\right)}{\sqrt{\frac{1}{{\sigma_{1}}^{2}}{\overset{-}{A}}_{1j}\left(f\right){{\overset{-}{A}}_{1j}}^{H}\left(f\right)+\frac{1}{{\sigma_{2}}^{2}}{\overset{-}{A}}_{2j}\left(f\right){{\overset{-}{A}}_{2j}}^{H}\left(f\right)}}$$ (1)

The denominator in Equation 1 serves as a normalization factor that constrains gPDC coefficients to the range of 0–1. The choice of scaling means that $\left|{\pi_{ij}}^{g}\left(f\right)\right|$ measures the outflow of information from signal $x_{j}$ to signal $x_{i}$ with respect to the total outflow of information from $x_{j}$ to all signals.

### 2.11 Data and statistical analysis

**FIGURE 1:**
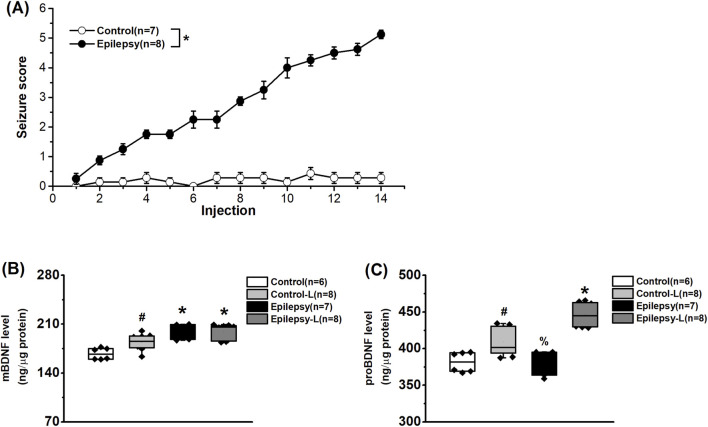
Seizure scores and hippocampal BDNF/proBDNF expression in PTZ-induced epileptic rats. **(A)** Seizure scores following PTZ induction. Rats were administered PTZ 14 times at a dose of 37 mg/kg *, *P* < 0.05, vs. control. **(B)** Hippocampal mBDNF levels in control and PTZ-induced epilepsy groups, and their expression following the behavioral training. **(C)** The proBDNF levels in control and PTZ-induced epilepsy groups, and their expression following the behavioral training. #, *P* < 0.05, vs. control; *, *P* < 0.05, vs. control and control-L; %, *P* < 0.05, vs. epilepsy-L. The number of rats in each group was indicated in figure legend.

**FIGURE 2:**
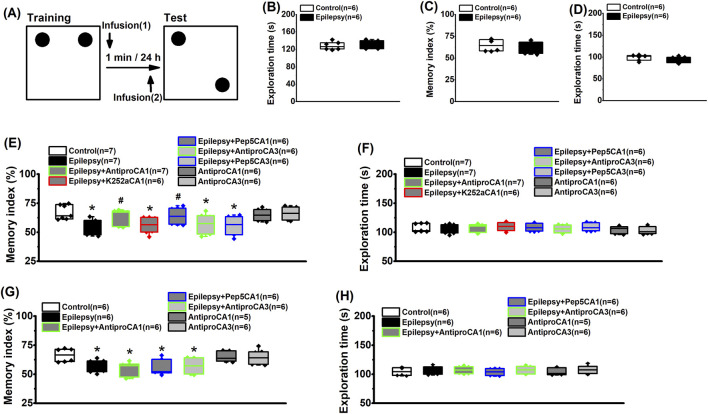
The performance in the behavioral training and memory test. **(A)** The diagram illustrating an OPR task and experimental procedure. The memory tests were conducted immediately following the training (STM) or 24 h after the training (LTM). In the LTM test, the infusions were conducted immediately following the training (Infusion 1) or 30 min before the memory testing (Infusion 2) to evaluate the effects on memory consolidation and retrieval stages, respectively. **(B)** Total exploration time during the training stage. Memory index **(C)** and total exploration time **(D)** during the STM test. Memory index **(E)** and total exploration time **(F)** during the LTM test when the intra-hippocampal infusions were conducted immediately following the training. #, *P* < 0.05, vs. epilepsy; *, *P* < 0.05, vs. control, epilepsy + AntiproCA1, epilepsy + Pep5CA1, AntiproCA1 and AntiproCA3. Memory index **(G)** and total exploration time **(H)** during the LTM test when the intra-hippocampal infusions were conducted 30 min before memory test. ACSF was infused into the CA1 region as the vehicle control for both Control and Epileptic groups; no ACSF control was applied to CA3, which was used only to test regional specificity. *, *P* < 0.05, vs. control, AntiproCA1 and AntiproCA3. The number of rats in each group was indicated in figure legend.

In this study, the hippocampal CA1 region was administered ACSF as a unified vehicle control for all CA1-targeted pharmacological interventions (anti-proBDNF antibody, TAT-Pep5, K252a) in both control and epileptic rats. The CA3 region was used solely to evaluate the regional specificity of proBDNF-p75^NTR^ signaling, and no additional ACSF vehicle control was performed in CA3. All pharmacological treatment groups were statistically compared against the ACSF-infused epileptic group (CA1) to assess intervention effects. All data were presented as Mean ± SEM. One-way and two-way ANOVA were used to analyze neural recording data, ELISA results and behavioral test outcomes, with subsequent Tukey’s *post hoc* test. Independent-sample t-test was employed to assess statistical difference between control and epilepsy groups (Figures 2B–D). Repeated-measures ANOVA was used to analyze seizure score (Figure 1A). All analyses were conducted using SPSS 21.0 software and the significant level was set at 0.05.

## 3 Results

### 3.1 The learning-induced mBDNF and proBDNF expression levels in the hippocampus

The repeat administration of subconvulsive doses of PTZ (37 mg/kg) every 2 days for a total of 14 injections produced chemical kindling. This phenotype was characterized by a progressive elevation in seizure score (Figure 1A, repeated-measure ANOVA, *F*_(1, 13)_ = 29.60, *P* < 0.001). The repeated PTZ treatment increased hippocampal mBDNF expression 24 h after the final PTZ treatment (Figure 1B; two-way ANOVA, treatment effect: *F*_(1, 27)_ = 6.57, *P* = 0.016; training effect: *F*_(1, 27)_ = 4.87, *P* = 0.036; interaction effects: *F*_(1, 27)_ = 6.01, *P* = 0.021; Epilepsy vs. Control, *P* < 0.05). In contrast hippocampal proBDNF levels were not altered in rats that exhibited convulsive seizures in response to the repeated PTZ injections (Figure 1C; two-way ANOVA, treatment effect: *F*_(1, 27)_ = 5.85, *P* = 0.023; training effect: *F*_(1, 27)_ = 8.89, *P* = 0.006; interaction effects: *F*_(1, 27)_ = 6.29, *P* = 0.018; Epilepsy vs. Control, *P* < 0.05). Following the behavioral training, both mBDNF and proBDNF expression were drastically upregulated in the control (mBDNF: Control-L vs. Control, *P* < 0.05; proBDNF: Control-L vs. Control, *P* < 0.05). However, the learning-induced mBDNF expression was not increased in epilepsy group (Epilepsy-L vs. Epilepsy, *P* > 0.05). In contrast the level of proBDNF in the epilepsy group was excessively enhanced following the behavioral training (Epilepsy-L vs. Epilepsy, *P* < 0.05). Therefore, these results indicate that behavioral learning triggers an abnormal increase in proBDNF without altering mBDNF levels in epilepsy rats.

### 3.2 Activation of proBDNF signaling is involves in spatial memory deficits in epilepsy

The OPR task is a well-validated method for studying learning and memory functions that depend on the hippocampal formation (Chao et al., 2020; Iwasaki et al., 2021; Yamada et al., 2017). PTZ kindling did not change exploration time during the learning period (Figure 2B; *t-test*, *t*_12_ = 0.21, *P* = 0.419). Immediately following the training (around 1 min), the short-term memory (STM) was tested and no statistical difference in memory index was observed between control and epilepsy groups (Figure 2C; *t-test*, *t*_10_ = 0.17, *P* = 0.434). Similarly PTZ treatment did not affect exploration behavior during the STM test (Figure 2D; *t-test*, *t*_10_ = 0.25, *P* = 0.403). In the memory test on the following day (long-term memory, LTM), PTZ-kindled rats that received ACSF infusion showed a significant decline in the memory index compared to non-kindled, ACSF-infusion control (Figure 2E; one-way ANOVA, *F*_(8, 48)_ = 3.89, *P* = 0.013; Epilepsy vs. Control, *P* < 0.05). Rats in the control and epilepsy groups received ACSF infusions into the hippocampal CA1 region as the unified vehicle control for all pharmacological interventions. All drug-treated groups were compared with the ACSF-infused epilepsy group to determine the effects of proBDNF/p75^NTR^ signaling blockade. Compared to the epilepsy group following ACSF infusion into CA1 area, blocking the increased proBDNF in hippocampal CA1 (Epilepsy-AntiproCA1 vs. Epilepsy, *P* < 0.05) but not CA3 (Epilepsy-AntiproCA3 vs. Epilepsy, *P* > 0.05) region could effectively enhance the declined memory index. The CA3 region received no independent ACSF control, as its role was limited to confirming the regional specificity of proBDNF function. Furthermore, to detect whether the proBDNF-induced deteriorative effect was mediated by p75^NTR^ or TrkB receptors, rats were subjected to training, followed by infusion of either Pep5 (p75^NTR^) or K252a (TrkB) selective antagonist into the CA1 area of PTZ-treated rats immediately following training (Figures 2E,F). As shown in Figure 2E, Epilepsy + K252aCA1 rats were indistinguishable from ACSF-infused epilepsy group in their memory index (Epilepsy + K252aCA1 vs. Epilepsy, *P* > 0.05). However, infusions of Pep5 into hippocampal CA1 but not CA3 region could significantly elevate the memory index compared to the ACSF-infused epilepsy group (Epilepsy + Pep5CA1 vs. Epilepsy, *P* < 0.05; Epilepsy + Pep5CA3 vs. Epilepsy, *P* > 0.05). To test the effect on memory retrieval stage, the infusions were conducted 30 min prior to memory test (Figures 2G,H). However, the decreased memory index in the ACSF-infused epilepsy group could not be reversed by anti-proBDNF antibody infusions into CA1 (Figure 2G, one-way ANOVA, *F*_(6, 35)_ = 3.85, *P* = 0.046; Epilepsy-AntiproCA1 vs. Epilepsy, *P* > 0.05) or CA3 (Epilepsy-AntiproCA3 vs. Epilepsy, *P* > 0.05), or p75^NTR^ inhibitor infusions into CA1 (Epilepsy-Pep5CA1 vs. Epilepsy, *P* > 0.05). Additionally, the time spent on exploration did not alter during LTM test (Figure 2F; one-way ANOVA, *F*_(8, 48)_ = 0.97, *P* = 0.470. Figure 2H; one-way ANOVA, *F*_(6, 35)_ = 0.82, *P* = 0.562). Therefore, these findings demonstrate that proBDNF -p75^NTR^, but not -TrkB, signaling is involved in proBDNF-induced memory impairments of PTZ-treated epilepsy rats.

### 3.3 Learning increases the activity of putative FS interneurons following PTZ induction

**FIGURE 3:**
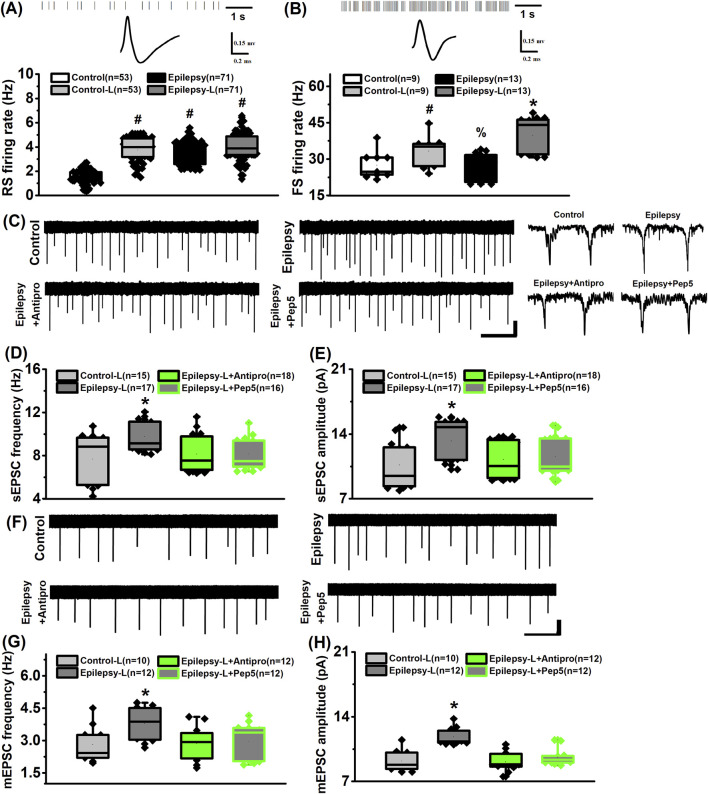
The changes in neuronal firing rates and EPSCs of CA1 putative FS interneurons. The firing rates of both RS **(A)** and putative FS **(B)** cells were significantly increased following behavioral training in the control animals while only the frequency of putative FS was abnormally increased after the training. Representative firing traces of RS pyramidal neurons and putative FS interneurons are shown as upper insets in panels **(A,B)**, respectively. #, *P* < 0.05, vs. control; *, *P* < 0.05, vs. control and control-L; %, *P* < 0.05, vs. epilepsy-L. **(C)** Representative traces of sEPSCs recorded from putative FS interneurons. A magnified segment of each trace is shown to clearly present individual sEPSC waveform and peak amplitude. Scale bars for sEPSCs: 0.5 s (horizontal) and 5 pA (vertical). Both frequency **(D)** and amplitude **(E)** of sEPSC were drastically enhanced following behavioral training in PTZ-treated epileptic animals. Incubation with anti-proBDNF antibody or TAT-Pep5 inhibitor could significantly suppressed the enhanced frequency and amplitude. **(F)** Representative traces of mEPSCs recorded from putative FS interneurons in the presence of TTX. Scale bars for mEPSCs: 0.5 s (horizontal) and 5 pA (vertical). **(G)** Mean frequency and **(H)** amplitude of mEPSCs. Both parameters were increased in the Epilepsy-L group and rescued by anti-proBDNF antibody or TAT-Pep5. *, *P* < 0.05, vs. others. The number of rats in each group was indicated in figure legend.

In the *in vivo* neuronal recording, data were obtained 53 pyramidal RS neurons and eight putative FS interneurons from six rats in the Control group, 53 pyramidal RS neurons and eight putative FS interneuron from six rats in the Control-L group, 71 pyramidal RS neurons and 13 putative FS interneuron from eight rats in the Epilepsy group, and 71 pyramidal RS neurons and 13 putative FS interneuron from eight rats in the Epilepsy-L group. The basal firing rate of hippocampal RS cells was significantly increased in the Epilepsy group (Figure 3A; two-way ANOVA, treatment effect: *F*_(1, 246)_ = 7.16, *P* = 0.008; training effect: *F*_(1, 246)_ = 5.56, *P* = 0.019; interaction effects: *F*_(1, 246)_ = 7.57, *P* = 0.006; Epilepsy vs. Control, *P* < 0.05), while the frequency of putative FS interneurons was not changed by the PTZ kindling (Figure 3B; two-way ANOVA, treatment effect: *F*_(1, 42)_ = 4.95, *P* = 0.031; training effect: *F*_(1, 42)_ = 7.93, *P* = 0.007; interaction effects: *F*_(1, 42)_ = 5.49, *P* = 0.023; Epilepsy vs. Control, *P* > 0.05). Learning could increase firing rate of both RS and putative FS cells in the Control group, while markedly increased frequency of putative FS interneurons in Epilepsy group (Epilepsy-L vs. Epilepsy, *P* < 0.05) was observed with no changes in RS neurons (Epilepsy-L vs. Epilepsy, *P* > 0.05).

In the separate treatment groups, to evaluate how PTZ kindling interfered with learning-mediated inhibitory transmission, spontaneous EPSCs were recorded in the putative FS interneurons from rats that performed behavioral training (Figure 3C). Recordings were obtained from 15 interneurons across six rats in the Control-L group, 17 interneurons across eight rats in the Epilepsy-L group, 18 interneurons across eight rats in the Epilepsy-L + Antipro group, and 16 interneurons across seven rats in the Epilepsy-L + Pep5 group. Both mean frequency (Figure 3D; one-way ANOVA, *F*_(3, 62)_ = 4.77, *P* = 0.005; Epilepsy-L vs. Control-L, *P* < 0.05) and amplitude (Figure 3E; one-way ANOVA, *F*_(3, 62)_ = 4.28, *P* = 0.008; Epilepsy-L vs. Control-L, *P* < 0.05) of sEPSCs were abnormally enhanced in the Epilepsy group, while anti-proBDNF and p75^NTR^ inhibitor Pep5 could effectively suppress the increased frequency (Epilepsy-L + Antipro vs. Epilepsy-L, *P* < 0.05; Epilepsy-L + Pep5 vs. Epilepsy-L, *P* < 0.05) and amplitude (Epilepsy-L + Antipro vs. Epilepsy-L, *P* < 0.05; Epilepsy-L + Pep5 vs. Epilepsy-L, *P* < 0.05). Therefore, these findings imply that the excessive proBDNF induced by PTZ can exaggerate the frequency and amplitude of sEPSCs in the interneurons, leading to impair neuronal function.

To delineate the precise synaptic loci of proBDNF action, we recorded mEPSCs in the presence of TTX. Representative mEPSC traces are shown in Figure 3F. Recordings were obtained from 10 interneurons across five rats in the Control-L group, 12 interneurons across six rats in the Epilepsy-L group, 12 interneurons across six rats in the Epilepsy-L + Antipro group, and 12 interneurons across six rats in the Epilepsy-L + Pep5 group. In the Epilepsy-L group, both the frequency (Figure 3G; one-way ANOVA, *F*_(3, 42)_ = 3.74, *P* = 0.018; Epilepsy-L vs. Control-L, *P* < 0.05) and amplitude (Figure 3H; one-way ANOVA, *F*_(3, 42)_ = 23.64, *P <* 0.001; Epilepsy-L vs. Control-L, *P* < 0.05) of mEPSCs were significantly increased compared to the Control-L group. Critically, these increases in mEPSC frequency and amplitude were both normalized by incubation with either the anti-proBDNF antibody (Epilepsy-L + Antipro vs. Epilepsy-L, *P* < 0.05) or the p75^NTR^ inhibitor TAT-Pep5 (Epilepsy-L + Pep5 vs. Epilepsy-L, *P* < 0.05). The elevation in mEPSC frequency indicates a proBDNF/p75^NTR^-dependent enhancement of the basal probability of quantally presynaptic release, while the increase in mEPSC amplitude provides definitive evidence for a potentiation of postsynaptic function.

### 3.4 PTZ kindling disrupts phase synchronization and information processing

**FIGURE 4:**
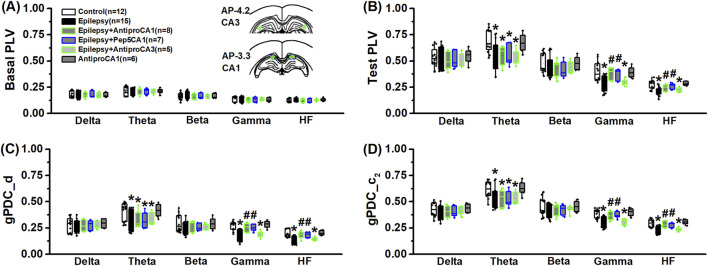
The interaction index of PLV and the directional coupling index *d* and *c*_*2*_ at the hippocampal CA3-CA1 synapses. **(A)** The basal values of PLV among groups are comparable. **(B)** During the memory test, the value of PLV of PTZ-induced epileptic animals was significantly declined in the theta, gamma and HF bands, but intra-CA1 infusions could effectively elevate the declined PLV value. **(C)** Coupling direction index *d* between CA1 and CA3 regions, indicating CA3 predominantly drives CA1. **(D)** Unidirectional influence index *c*_*2*_, indicating unidirectional information flow from CA3 to CA1 region. The values of *d* and *c*_*2*_ of PTZ-induced epileptic animals were declined while anti-proBDNF and P75^NTR^ inhibitor manipulation in the CA1 region could increase the values to the control levels. Rats in the Control and Epilepsy groups received intra-hippocampal CA1 infusions of ACSF as the vehicle control. No ACSF control was applied to the CA3 region, as CA3 was only used for verifying the regional specificity of proBDNF/p75^NTR^ signaling. *, *P* < 0.05, vs. control; #, *P* < 0.05, vs. epilepsy. The number of rats in each group was indicated in figure legend.

PTZ treatment and drug infusions did not have marked effect on the basal PLVs, which were recorded in the home-cage environment (Figure 4A, two-way ANOVA, treatment effect between treatment and band: *F*_(20, 188)_ = 0.69, *P =* 0.843). All comparisons were made relative to the ACSF-infused Epilepsy group (CA1), with no independent vehicle control in CA3. In contrast, compared to the control group with ACSF infusion into CA1 area, the PLVs of the ACSF-infused epilepsy group were significantly lower in theta, gamma and HF bands during the memory test (Figure 4B, two-way ANOVA, interaction effect between treatment and band: *F*_(20, 188)_ = 2.37, *P =* 0.001; Epilepsy vs. Control, all bands *P* < 0.05). However, compared to the epilepsy group with ACSF infusion into CA1 area, infusions of anti-proBDNF antibody into the hippocampal CA1 (Epilepsy + AntiproCA1 vs. Epilepsy, both *P* < 0.05) but not CA3 region (Epilepsy + AntiproCA3 vs. Epilepsy, both *P* > 0.05) could specifically elevate the declined gamma and HF PLVs. Meanwhile, inactivation of p75^NTR^ signaling in the CA1 region could also effectively reverse the decreased PLVs at gamma and HF bands (Epilepsy + Pep5CA1 vs. Epilepsy, both *P* < 0.05). To evaluate the NIF in the hippocampal CA3-CA1 pathway, the directionality index *d* of gPDC was calculated and compared. The directionality index *d* values of the ACSF-infused epilepsy group were drastically diminished at theta, gamma and HF bands compared to the ACSF-infused control group (Figure 4C; two-way ANOVA, interaction effect between treatment and band: *F*_(20, 188)_ = 2.19, *P* = 0.004; Epilepsy vs. Control, all bands *P* < 0.05). Furthermore, the values of the unidirectional influence c_2_ of the ACSF-infused epilepsy group, indicating the unidirectional coupling from CA3 to CA1, was significantly declined at the same bands compared to the ACSF-infused control group (Figure 4D; two-way ANOVA, interaction effect between treatment and band: *F*_(20, 188)_ = 2.06, *P* = 0.007; Epilepsy vs. Control, all bands *P* < 0.05). Similarly, compared to the ACSF-infused epilepsy group, blocking the excessive proBDNF or inhibition of p75^NTR^ signaling in the CA1 area could mitigate the weakened NIF, as the indicated by the elevated d (Epilepsy + AntiproCA1 vs. Epilepsy, both *P* < 0.05) and c_2_ (Epilepsy + Pep5CA1 vs. Epilepsy, both *P* < 0.05) values at both gamma and HF bands. However, infusions of anti-proBDNF antibody into the CA3 region could not rescue the declined d (Epilepsy + AntiproCA3 vs. Epilepsy, both *P* > 0.05) or c_2_ (Epilepsy + Pep5CA3 vs. Epilepsy, both *P* > 0.05) values. Additionally, we did not observe side effects of the CA1 infusions on basal PLV, test PLV, index d or c2 of gPDC. Together, these findings indicate that PTZ kindling suppresses the hippocampal NIF from CA3 to CA1, and proBDNF and its p75^NTR^ signaling over-activation is crucial for the declined NIF, especially high-frequency oscillations.

## 4 Discussion

To our knowledge, the present study provides the first direct evidence that learning-induced abnormally elevated proBDNF expression in the hippocampus of PTZ-kindled epilepsy rats. We further identified evidence that this increased proBDNF expression contributes to impairments in spatial memory consolidation. Blocking proBDNF or inhibiting p75^NTR^ signaling in the hippocampal CA1 region but not CA3 region of PTZ-kindled animals effectively mitigates memory deficits. Meanwhile, this learning-induced proBDNF can markedly facilitate the firing rate of putative FS interneurons by enhancing frequency and amplitude of the EPSCs. Furthermore, infusions of anti-proBDNF antibody and p75^NTR^ inhibitor into the CA1 area can effectively promote phase synchronization and elevate the directional index of NIF in PTZ-treated rats during memory testing. Collectively, these data suggest that learning-induced over-expression of the proBDNF and its activated signaling represent potential mechanisms underlying neural dysfunction and NIF disruption, ultimately leading to impairments in memory consolidation in the PTZ-kindled epilepsy model.

Several research groups have previously assessed BDNF levels in epilepsy models (Chen and Chen, 2017; Falcicchia et al., 2018; Parrish et al., 2015; Sun Z. Q. et al., 2019; Lin et al., 2020). Most of these studies quantified mRNA or mBDNF levels, but did not determine the levels of proBDNF. A few studies have evaluated proBDNF and mBDNF levels acutely after status epilepticus induction. They reported proBDNF increases as early as 3 h after status epilepticus onset with levels remaining elevated 24 h post-status epilepticus (Thomas et al., 2016). Elevated proNGF protein levels have also been observed 24 h after kainate-induced seizures *in vivo* (Le and Friedman, 2012; Volosin et al., 2008). This increase in proNGF was not accompanied by rises in mature NGF and resulted from inhibition of proneurotrophin cleavage (Le and Friedman, 2012). Most recently, one study reported that both proBDNF and mBDNF levels were elevated 24 h after a single dose of pilocarpine-induced status epilepticus in mice (VonDran et al., 2014). In contrast other researchers analyzed BDNF levels after pilocarpine-induced status epilepticus in adult rats and showed no changes in proBDNF immunoreactivity 3 days post-status epilepticus (Ullal et al., 2007; Mizoguchi et al., 2011). These findings indicate that rapid proBDNF increases following status epilepticus partially result from reduced cleavage. They also suggest proBDNF may be part of the initial neurotrophin response that drives intracellular signaling during the acute phase of epileptogenesis (Thomas et al., 2016; Mizoguchi et al., 2011). This hypothesis is supported by the latest findings that increased proBDNF synthesis and processing associated with kindling may maintain steady-state proBDNF levels while elevating hippocampal mBDNF levels (Thomas et al., 2016; Segawa et al., 2013).

Training-mediated proBDNF expression is considered as an important modulator of neural transmission and memory processes (Gibon et al., 2015; An et al., 2018; Sun et al., 2018a). Several studies suggest learning-induced proBDNF secretion that appears to be mediated at least in part by p75^NTR^ receptors activation, which can promote neural correlates with cognitive performance such as the formation of fear memory and spatial learning strategies (An et al., 2018; Ma et al., 2022; Sun W. et al., 2019). However, limited proBDNF cleavage capacity or excessively increased expression can cause both learning-induced and exogenous proBDNF to trigger aberrant synaptic pruning and exaggerated synaptic depression, leading to weaken synaptic connections in memory encoding (Je et al., 2012; Deinhardt and Chao, 2014; Gibon et al., 2017). Furthermore, endogenous proBDNF can regulate training-induced phosphorylation of N-methyl-D-aspartate (NMDA) receptor GluN2B subunits, which weaken memory traces and initiate memory destabilization (Sun et al., 2022a). Consistent with the NMDA-dependent dual role of proBDNF in developing synapses and synaptic plasticity (Langlois et al., 2013; Sun et al., 2022d; Gibon et al., 2017), our findings extend these observations and indicate proBDNF exerts bidirectional regulation of neuronal and cognitive function. Additionally, other neurodegenerative studies have found that spatial training increases proBDNF metabolism in aged and schizophrenic rats (Sun et al., 2022e; Buhusi et al., 2017; Chen et al., 2017). Consistently, hippocampal proBDNF over-expression induces deficits in neuronal morphology and spine density, ultimately leading to neuronal and synaptic dysfunction (Sun et al., 2022e; Xue et al., 2022).

In this study, we confirmed that the over-expression of proBDNF in the CA1 but not the CA3 region contributes to memory impairments. Consistently, reduced inhibitory function in the CA1 region may impact memory traces (Jeong and Singer, 2022; Geiller et al., 2023). Molecular signaling within the CA1 has also been identified as critical for recognition memory (Asgeirsdottir et al., 2020; Cinalli et al., 2020). In adult rats, epileptic seizures primarily affect CA1 neurons in comparison to other hippocampal subregions (Siddiqui and Joseph, 2005) while neurons in the CA3 subregion exhibited the highest resistance to PTZ-induced seizures (Vasilev et al., 2018). Our results align with previous findings that the hippocampal CA1 rather than other regions is selectively targeted by PTZ treatment and related psychotic disorders (Pasquetti et al., 2019; Lopez-Santiago et al., 2017). Thus, it is plausible to hypothesize that CA1 hypermetabolism may drive dysfunction in other brain regions fully developed epileptic conditions.

Abnormalities in inhibitory synaptic neurotransmission have been linked to epileptic activity (Serratto et al., 2020; Sears and Hewett, 2021; Dai et al., 2021). Our study showed that learning-induced proBDNF in PTZ-treated animals can exaggerate the excitability of putative FS interneurons in the CA1 region. ProBDNF has previously been found to acutely modulate the paired-pulse ratio and the frequency of mEPSCs (Sun et al., 2022a; Gibon et al., 2016), all indicating a presynaptic effect of proBDNF. At the postsynaptic level, proBDNF promotes phosphorylation of GluN2B subunits and enhances amplitude of sEPSCs (Sun et al., 2021a; Mizui et al., 2015). Our synaptic analyses reveal a multifaceted mechanism by which proBDNF/p75^NTR^ signaling strengthens excitatory input to FS interneurons. The increase in sEPSC frequency reflects enhanced presynaptic release ​triggered by action potentials. The mEPSC data provide further insight, showing that this presynaptic enhancement involves an increased basal probability of quantal release, as indicated by elevated mEPSC frequency, which may result from changes in presynaptic machinery or stability. Concurrently, the rise in mEPSC amplitude offers clear evidence of potentiated postsynaptic AMPA receptor function. Thus, proBDNF acts in concert to enhance synaptic transmission through multiple pathways, raising the intrinsic spontaneous release from presynaptic terminals, improving the reliability of action potential-evoked release, and strengthening the postsynaptic response. This comprehensive synaptic reinforcement provides a robust cellular foundation for the hyperexcitability of FS interneurons. Crucially, this increased activity of FS interneurons accompanied by unchanged RS firing in kindled animals after learning suggests an altered excitation-inhibition balance. ProBDNF/p75^NTR^-dependent enhancement of excitatory synaptic drive onto FS interneurons likely elevates inhibitory output, which may restrain further increases in RS excitability and contribute to the disrupted neural information flow at CA3-CA1 synapses. Functional blockade of p75^NTR^ during the latent phase after status epilepticus significantly reduces ictal discharges during the chronic phase of the pathology and restores the γ-aminobutyric acid (GABA) excitatory/inhibitory developmental sequence (Riffault et al., 2018). Importantly, our results showed that functional inhibition of p75^NTR^ mitigates the upregulated frequency and amplitude of putative FS EPSCs. This was in line with previous studies where rats infused with the p75^NTR^ inhibitor TAT-Pep5 displayed decreased pyramidal neuron firing propensity, indicating that the proBDNF-p75^NTR^ axis regulates neuroexcitability and persistent activity to balance neural excitation and inhibition (Gibon et al., 2015). Moreover, functional changes in proBDNF-mediated neuronal responses under developmental and pathological conditions parallel changes in the expression levels of p75^NTR^ but not TrkB (Sun et al., 2021a; Yang et al., 2022; Savall et al., 2023). Since activation of p75^NTR^ signaling is found to be upregulated in apoptotic neurons after seizures (Friedman, 2010; Malik et al., 2021), it is not surprising that proneurotrophins are particularly relevant to epilepsy. Altogether, our data suggest that the negative action of proBDNF on PTZ-induced neuronal hyperexcitability is linked to p75^NTR^ signaling.

Synchronization patterns change dynamically with stimulation and behavioral context. This dynamic change strongly suggests that selective coherence enables selective cell communication (Grover et al., 2021; Fries, 2015). As the major form of BDNF released in response to physiological stimuli, proBDNF is considered to play a key role in regulating information processing by modifying the excitation threshold in neural networks (Gibon et al., 2016). For example, enhancement in proBDNF-mediated neural coupling reflects stabilization of striatum-dependent reversal learning and promotes subthreshold low-frequency induced synaptic depression (Sun et al., 2020). Similarly, a previous study has demonstrated that high-frequency neuronal activity can activity-dependently control the opposing functions of BDNF isoforms by facilitating the conversion of proBDNF to mBDNF (Nagappan et al., 2009), which is critical for inducing synaptic potentiation and memory consolidation (Pang et al., 2004; Notaras et al., 2020; Pang et al., 2016). Actually, synaptic plasticity alters the strength of information flow between presynaptic and postsynaptic neurons, thereby regulating the probability that action potentials in presynaptic neurons trigger action potentials in postsynaptic neurons (Kallupi et al., 2014). Our results showed that both frequency and amplitude of EPSCs in the CA1 region declined in PTZ-treated animals, suggesting the disruption of presynaptic and postsynaptic information processing (Shore et al., 2020; Meier et al., 2014; Chen et al., 2020). Additionally, the depolarizing actions of GABA in the cortex of proBDNF-treated animals enhanced neuronal network activity, leading to impairments in synaptic efficacy and memory-related behavior (Riffault et al., 2018; Chun et al., 2018) and has the potential to disturb ongoing information processing (Dai et al., 2021; Park and Poo, 2013; Aljadeff et al., 2021). Emerging evidence indicates that gamma oscillatory activity is heterogeneous during learning and memory with variations in the strength of gamma power (Mably and Colgin, 2018; Kanta et al., 2019). Furthermore, gamma and HF synchronization are generated by inhibitory putative FS interneurons and reductions in these synchronizations theoretically reflect decreased phasic inhibition, a characteristic assumed to define this epileptic phenotype (Barron, 2021; Khazipov, 2016; Shuman et al., 2020). Our latest studies suggest that proBDNF-mediated neural oscillations play a prominent role in preventing fear memory processing, with particular emphasis on facilitation of memory destabilization and extinction (Sun et al., 2022a; Sun W. et al., 2019). Our current study is the first to demonstrate that disrupted neural synchronization between hippocampal CA3 and CA1 regions in PTZ-treated rats leads to impairments in memory formation. This finding complements the observed weakening of CA3-CA1 NIF at higher frequencies, including gamma and HF bands.

Several potential factors may explain why we detected proBDNF increases not reported in previous reported. These factors include differences in species, background strain, and model of status epilepticus (Le and Friedman, 2012; Thomas et al., 2016; Segawa et al., 2013). Repeating these studies using identical techniques across different models would help clarify how model selection impacts findings. Although we found that proBDNF could strengthen the direction of NIF during higher-frequency oscillations, theta oscillations, which enhance spatial memory processes (Herweg et al., 2020; Kragel et al., 2020), were not markedly changed in the CA3-CA1 pathway. Indeed, gamma oscillations are known to nest within the theta oscillations during memory tasks (Rayan et al., 2022; Lopez-Pigozzi et al., 2016). The disparities observed here may therefore reflect changes in cross-frequency coupling. The mechanisms underlying this effect require further investigation.

Furthermore, the present study focused on elucidating the effect of proBDNF/p75^NTR^ signaling on excitatory synaptic transmission onto FS interneurons, which we identified as a key driver of network imbalance. Future investigations examining the potential direct modulation of synaptic transmission in RS pyramidal neurons by this pathway will be valuable to provide a more complete mapping of its actions within the hippocampal CA1 microcircuit.

However, the sample sizes employed in behavioral and electrophysiological experiments are moderate and no formal *a priori* statistical power analysis was performed to determine group sizes. The use of multiple ANOVA-based comparisons across various datasets without additional stringent correction for multiple testing may increase the risk of both Type I (false-positive) and Type II (false-negative) statistical errors. Accordingly, the present findings should be interpreted with appropriate caution. The present study was designed to investigate network and synaptic level dysfunctions, and thus does not directly address whether proBDNF/p75^NTR^ signaling influences intrinsic action potential properties of hippocampal interneurons, such as depolarization/repolarization kinetics or rheobase. It is noteworthy that modulation of intrinsic excitability by proBDNF/p75^NTR^ signaling represents a plausible mechanism supported by emerging literature (Gibon et al., 2015; Gibon et al., 2016). For instance, p75^NTR^ activation has been linked to regulation of voltage-gated potassium channels, which are critical determinants of action potential repolarization and firing frequency (Deinhardt and Chao, 2014; Gibon et al., 2017). Moreover, proBDNF has been shown to alter intrinsic excitability in cortical pyramidal neurons (Gibon et al., 2016). Notably, the p75^NTR^ receptor itself is implicated in the pathophysiology of epilepsy, where it may contribute to network dysregulation (Turkistani et al., 2024). In epilepsy, aberrations in intrinsic neuronal properties are a well-established hallmark of network hyperexcitability (Royer et al., 2022; Rho and Boison, 2022). Future studies using current-clamp recordings from identified FS interneurons in hippocampal slices, combined with selective manipulation of the proBDNF/p75^NTR^ pathway, will be necessary to directly test this hypothesis and to dissect the relative contributions of synaptic versus intrinsic mechanisms to network dysfunction in this PTZ kindling model.

Our findings provide the first evidence that learning-induced proBDNF changes severely disrupt neural function in the epileptic hippocampus and contribute to epilepsy-associated memory deficits, particularly in memory consolidation. Therefore, dysfunction in proBDNF-mediated inhibition can lead to neuronal network hyperexcitability and the emergence of cognitive deficits in neurological disorders. Collectively, these data highlight the pivotal role of proBDNF signaling in memory formation and may explain some of the mechanisms that could inform new therapeutic strategy to prevent memory loss in epilepsy.

## Data Availability

All data supporting the results of this study are publicly available at Figshare: https://doi.org/10.6084/m9.figshare.32287950. The data that support the findings of this study are available from the corresponding author upon reasonable request.
